# Successful Treatment of Whole Coronary Artery Spasm after Off-Pump Coronary Artery Bypass Grafting

**DOI:** 10.1155/2022/9003921

**Published:** 2022-09-09

**Authors:** Akihiro Kobayashi, Yoshimori Araki, Takafumi Terada, Osamu Kawaguchi

**Affiliations:** Department of Cardiac Surgery, Toyota Kosei Hospital, Ibobara 500-1, Jousui-cho, Toyota, Aichi 470-0396, Japan

## Abstract

Coronary artery spasm after coronary artery bypass grafting is a rare but life-threatening condition. Herein, we report the case of a 77-year-old man who received off-pump coronary artery bypass grafting. An hour after surgery, there was a sudden hemodynamic compromise due to coronary artery spasm, prompting emergent coronary angiography with extracorporeal membrane oxygenation support. Because the angiography results showed diffuse severe spasm of the entire native coronary artery, the patient was treated with an intracoronary injection of vasodilators. The patient recovered in 7 days with mechanical support, catecholamines, and vasodilators, and he was discharged on postoperative day 30. Although coronary artery spasm after off-pump coronary artery bypass surgery is a rare condition, it must be suspected when sudden circulatory collapse occurs.

## 1. Introduction

Coronary artery spasm following off-pump coronary artery bypass surgery (OPCAB) is relatively rare but can lead to serious complications, including death [1]. Herein, we report a case of coronary artery spasm after OPCAB, which was successfully treated with percutaneous cardiopulmonary support, urgent coronary angiography, and intracoronary vasodilator infusion.

## 2. Case Presentation

**Figure 1:**
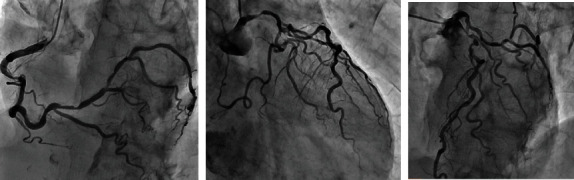
Coronary angiography showed a 75% stenosis of the left main coronary artery, 99% stenosis of the left ascending artery, 90% stenosis of the right coronary artery, and 75% stenosis of the atrioventricular branch.

**Figure 2:**
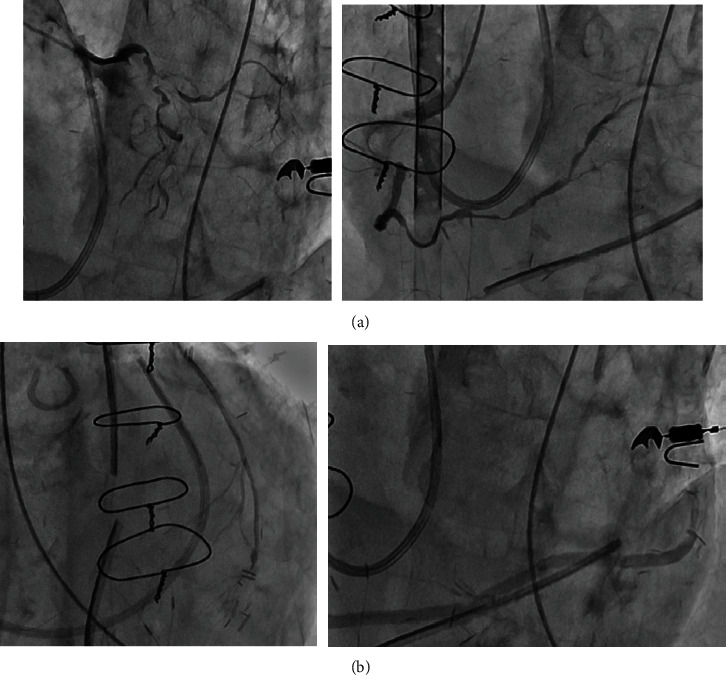
Emergent coronary angiography. Diffuse severe spasm of whole native coronary arteries (a). The internal mammary artery and great saphenous vein were patent (b).

**Figure 3:**
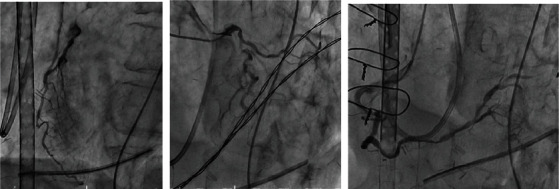
Coronary spasm was still partially present, but flow gradually improved.

A 77-year-old man had a chief complaint of chest pain at rest and was referred to our hospital with a diagnosis of unstable angina pectoris. Coronary angiography (CAG) showed 75% stenosis of the left main coronary artery and 90% stenosis of the right coronary artery. Although coronary artery bypass grafting was recommended, the patient did not agree with the operation. Instead, he opted to take medications, including aspirin, carvedilol, nicorandil, beraprost sodium, and isosorbide dinitrate. Six months later, he was admitted to our hospital because of the increasing frequency of chest pain and agreed to undergo surgery. Preoperative CAG showed 75% stenosis of the left main coronary artery and 99% stenosis of the left ascending artery (LAD), which was worse compared to the patient's condition 6 months ago (90% stenosis of the right coronary artery and 75% stenosis of the atrioventricular branch) (Figure 1). An intra-aortic balloon pump (IABP) was placed because of chest pain, ST depression on electrocardiography (ECG), and elevation of blood pressure during CAG. Following IABP placement and vasodilator infusion, the patient's symptoms disappeared. Three days later, we performed OPCAB, which was selected as the treatment method because of the highly calcified ascending aorta. Initially, the patient underwent a left internal mammary artery (LIMA) anastomosis to the LAD and a sequential great saphenous vein anastomosis to the posterior descending branch and atrioventricular branch. He returned to the intensive care unit (ICU) in a stable condition while on noradrenaline (0.07 *μ*g/kg/min), dobutamine (2 *μ*g/kg/minute), and nicorandil. An hour later, his heart rate and systolic blood pressure immediately decreased to 40 bpm and 40 mmHg, respectively. Moreover, his cardiac index (CI) dropped from 2.2 to 0.9, and his pulmonary artery pressure (PAP) and central venous pressure (CVP) increased. Hemodynamics improved after temporary pacing and increased administration of inotropes and volume resuscitation; however, the ECG showed no ST changes. Despite minimal bleeding from the drain, we suspected cardiac tamponade and decided to reopen the chest. While preparing for the surgery, his heart rate suddenly decreased again and self-pulse disappeared, which prompted chest reopening in the ICU. On inspection, there was minimal hematoma, there were no signs of tamponade, and there were no signs of aortic dissection grossly or on the transesophageal echocardiogram (TEE). Hemodynamics subsequently improved, and the blood pressure increased to 90 mmHg with the administration of adrenaline; therefore, the chest was closed. At that time, his PAP was 35/25 mmHg, and his CVP was 30 mmHg. Fifteen minutes after reclosing the chest, his heart rate dropped once again, but temporary pacing was no longer responsive. Extracorporeal membrane oxygenation (ECMO) was immediately started, and the patient was transported to the cardiac catheterization laboratory. During this process, the ECG monitor showed ST elevation in lead II, and emergent CAG revealed patent grafts and diffuse severe spasms of the whole native coronary arteries (Figure 2). The right and left coronary arteries were directly infused with sodium nitroprusside hydrate, nicorandil, and isosorbide dinitrate. The ST elevation in V1–6 became clearer after the direct infusion of the drug into the right coronary artery. We also administered the aforementioned drugs into the LIMA and saphenous vein grafts. As a result, coronary flow gradually improved, and the blood pressure increased (Figure 3). Although coronary spasms of the left circumflex artery, right coronary artery, and LAD were still partially present, hemodynamics and ST elevation improved. The patient was then returned to the ICU, and nicorandil and nitroglycerin drips were continued. Peak creatinine phosphokinase and MB isozyme concentrations on postoperative day 1 were 8470 IU/L and 769 U/L, respectively. On postoperative day 3, ECMO was discontinued with dobutamine (4 *μ*g/kg/min) and milrinone (0.2 *μ*g/kg/min). On postoperative day 7, IABP was discontinued with dobutamine (4 *μ*g/kg/min) and milrinone (0.25 *μ*g/kg/min), and the patient was extubated on the next day. On postoperative day 9, his hemodynamics remained stable even without inotropes, and he was discharged from the ICU on postoperative day 30.

## 3. Discussion

As observed in our case, sudden hemodynamic collapse after coronary artery bypass may be due to coronary artery spasm. Although relatively rare, coronary artery spasms can lead to serious complications, including death, with a postoperative mortality of 10%–50%. The incidence of coronary artery spasm following cardiac surgery has been reported to range from 0.8% to 1.3%; however, this may be underestimated since its diagnosis requires coronary angiography [1]. From April 2002 to November 2021, a total of 1612 cardiac surgeries were performed at our institution. Among them, 936 were isolated CABG cases and 395 were OPCAB cases. Notably, there were two cases of postoperative coronary artery spasm following OPCAB, suggesting an incidence rate of 0.51%. A study reported that in a normal RCA without stenosis, coronary artery spasm commonly develops after weaning from cardiopulmonary bypass within 2 h after surgery [2]. Currently, the etiology of postoperative coronary artery spasm is unclear, but several factors have been reported to induce this condition. Smoking is a major risk factor for coronary artery spasm in the absence of significant coronary stenosis. Minato et al. reported that hypomagnesemia is one of the triggers of coronary artery spasm, which is widespread in OPCAB [3]. This is why magnesium is included in the cardioplegia solution for on-pump CABG because of its protective effect against coronary artery spasm. Preoperative use of beta-blockers and calcium blockers, trauma due to surgical manipulation, hypothermia, hyperventilation, and compression by chest tubes have also been reported as etiologic factors of postoperative coronary arterial spasm [4, 5]. This is consistent with our case, since the patient had a history of smoking and medication with preoperative beta-blockers. CAG is the gold standard for diagnosing coronary artery spasms; however, this may become apparent clinically through ECG changes, hemodynamic instability, arrhythmia, circulatory collapse, or cardiac arrest [1]. Among these, the most common manifestations are ST-segment elevation and circulatory collapse without a specific cause. In our case, ECG changes were not apparent, and the patient developed circulatory collapse. In patients with unstable hemodynamics, the use of an IABP can increase coronary flow and support the left ventricle. However, in cases of severe cardiogenic shock or cardiac arrest, an IABP is not enough to support the patient's hemodynamics. Instead, more advanced mechanical circulatory supports, such as venoarterial ECMO, should be performed. Particularly, ECMO is reported to provide full circulatory support, such that catecholamine infusions that may aggravate cardiac artery spasm by the alpha-adrenergic effect can be discontinued. Moreover, ECMO improves oxygenation, decompresses the heart, and reduces myocardial oxygen consumption, thereby reducing any ongoing ischemia. Once stabilized on ECMO, the patient can be safely transported to the catheterization laboratory [1]. In our case, circulatory collapse suddenly occurred, making immediate diagnosis difficult. As the first priority was to stabilize the circulation, we first inserted the TEE and reopened the chest, allowing us to rule out tamponade and dissection. ECMO was then performed since the patient's hemodynamics did not improve. After the circulation was stabilized, CAG was performed, thereby allowing the diagnosis of coronary artery spasm and treatment with direct injection of vasodilators into the coronary arteries. The trigger in this case was unclear, but hypomagnesemia was noted. Specifically, the preoperative magnesium level was 1.7 mg/dL, but the postoperative level was 1.4 mg/dL. Therefore, hypomagnesemia could be considered a risk factor of coronary artery spasm in this case. Moreover, although magnesium is replenished from cardioplegia in conventional on-pump CABG, the OPCAB value of magnesium should still be monitored carefully.

In conclusion, we report a case of hemodynamically significant coronary artery spasm after OPCAB that was successfully treated with ECMO and intracoronary infusion of vasodilators. Although coronary artery spasm after OPCAB is rare, it should still be suspected when sudden circulatory collapse occurs.

## Data Availability

Data are available upon request.
